# The role of CXC-chemokine receptor CXCR2 and suppressor of cytokine signaling-3 (SOCS-3) in renal cell carcinoma

**DOI:** 10.1186/1471-2407-14-149

**Published:** 2014-03-04

**Authors:** Anastasios Stofas, Georgia Levidou, Christina Piperi, Christos Adamopoulos, Georgia Dalagiorgou, Aristotelis Bamias, Alexandra Karadimou, George A Lainakis, Stefanos Papadoukakis, Konstantinos Stravodimos, Meletios-Athanasios Dimopoulos, Efstratios Patsouris, Hariklia Gakiopoulou, Penelope Korkolopoulou

**Affiliations:** 1 First Department of Pathology, Laiko Hospital, University of Athens, Medical School, 75 Mikras Asias street, 11527 Athens, Greece; 2 Department of Biological Chemistry, University of Athens, Medical School, 11527 Athens, Greece; 3 Department of Clinical Therapeutics, Alexandra General Hospital, University of Athens, Medical School, 11528 Athens, Greece; 4 Department of Urology, Laiko Hospital, University of Athens, Medical School, 11517 Athens, Greece

**Keywords:** CXCR2, SOCS-3, IL-6, IL-8, Microvessels, Angiogenesis, Renal cell carcinoma

## Abstract

**Background:**

Chemokine receptor signaling pathways are implicated in the pathobiology of renal cell carcinoma (RCC). However, the clinical relevance of CXCR2 receptor, mediating the effects of all angiogenic chemokines, remains unclear. SOCS (suppressor of cytokine signaling)-3 is a negative regulator of cytokine-driven responses, contributing to interferon-α resistance commonly used to treat advanced RCC with limited information regarding its expression in RCC.

**Methods:**

In this study, CXCR2 and SOCS-3 were immunohistochemically investigated in 118 RCC cases in relation to interleukin (IL)-6 and (IL)-8, their downstream transducer phosphorylated (p-)STAT-3, and VEGF expression, being further correlated with microvascular characteristics, clinicopathological features and survival. In 30 cases relationships with hypoxia-inducible factors, i.e. HIF-1a, p53 and NF-κΒ (p65/RelA) were also examined. Validation of immunohistochemistry and further investigation of downstream transducers, p-JAK2 and p-c-Jun were evaluated by Western immunoblotting in 5 cases.

**Results:**

Both CXCR2 and IL-8 were expressed by the neoplastic cells their levels being interrelated. CXCR2 strongly correlated with the levels of HIF-1a, p53 and p65/RelA in the neoplastic cells. Although SOCS-3 was simultaneously expressed with p-STAT-3, its levels tended to show an inverse relationship with p-JAK-2 and p-c-Jun in Western blots and were positively correlated with HIF-1a, p53 and p65/p65/RelA expression. Neither CXCR2 nor SOCS-3 correlated with the extent of microvascular network. IL-8 and CXCR2 expression was associated with high grade, advanced stage and the presence/number of metastases but only CXCR2 adversely affected survival in univariate analysis. Elevated SOCS-3 expression was associated with progression, the presence/number of metastasis and shortened survival in both univariate and multivariate analysis.

**Conclusions:**

Our findings implicate SOCS-3 overexpression in RCC metastasis and biologic aggressiveness advocating its therapeutic targeting. IL-8/CXCR2 signaling also contributes to the metastatic phenotype of RCC cells but appears of lesser prognostic utility. Both CXCR2 and SOCS-3 appear to be related to transcription factors induced under hypoxia.

## Background

Renal cell carcinoma (RCC) is notorious for its angiogenic properties [1,2] and its ability to evade immunosurveillance. Therefore, not surprisingly, current literature is replete with studies looking into the mechanisms underlying the angiogenic phenotype of this tumor. The vast majority of these tumors are associated with the inactivation of the von Hippel-Lindau (VHL) tumor suppressor gene, which leads to the stabilization of hypoxia-inducible factor-1α (HIF-1α) with consequent enhanced transcription of many proangiogenic factors, such as vascular endothelial growth factor (VEGF) [3].

Cytokines are major regulatory proteins controlling the growth and differentiation of normal and malignant cells and contributing to the immune system’s failure to recognize tumor cells [4]. The pleiotropic cytokine interleukin (IL)-6, in particular, is known to induce the expression of VEGF [5], which is considered to be a major endothelial mitogen in RCC. IL-6 is one of the most ubiquitously deregulated cytokines in cancer, including RCC (rev. in [6]). IL-6 signals through a cell surface type I cytokine receptor including the signal transducing component GP130 which activates the tyrosine kinase JAK and ultimately the signal transducer and activator of transcription (STAT)-3 [7]. The latter is activated through phosphorylation at Tyr^705^ also in response to growth factors and extracellular signals [8]. Once phosphorylated, STAT-3 translocates to the nucleus where it binds to IFN-γ-activated site-like DNA elements [9], inducing the expression of genes promoting abnormal cell cycle progression, angiogenesis, inhibition of apoptosis, tissue invasion and immune evasion [10].

Chemokines are small chemoattractant cytokines that play an integral role in the pathobiology of RCC (rev. in ref [4]). The ELR + family of CXC-chemokines to which interleukin (IL)-8 belongs are recognized as potent promoters of angiogenesis by virtue of the Glu-Leu-Arg (ELR) motif immediately preceding their first N-terminal cysteine residue [11]. IL-8 effects are mediated by two highly related G-protein coupled receptors – chemokine (C-X-C motif) receptor 1 (CXCR1) and CXCR2. CXCR2 is promiscuous in nature since it can bind all other ELR + chemokines [12] and constitutes the prime functional chemokine receptor mediating endothelial cell chemotaxis in response to ligand binding [13]. Recently, IL-8 has been identified as a contributor to resistance to the anti-angiogenic agent sunitinib in RCC [14].

The interest in the identification of up-stream regulators of the cytokine-driven STAT activation stems from the profound biologic consequences of uncontrolled cytokine signaling [15]. To date, the only known inhibitors in this regard are the suppressors of cytokine signaling (SOCS), comprising SOCS-1–SOCS-7 and the cytokine-inducible SH2 domain containing protein. These proteins recognize phosphorylated tyrosine residues on JAKs and/or cytokine receptor subunits, thereby attenuating response to cytokines or growth factors [16]. STAT-3 induces SOCS-3 which feeds back to negatively regulate JAK/STAT [17]. Due to their rapid induction and quick turnover, SOCS proteins act as negative regulators of IFN-α signaling by inhibiting the JAK/STAT pathway, thereby opposing its proliferative and anti-apoptotic and apoptotic effect [18]. However, the function of SOCS is more complex than originally thought since they may facilitate or suppress neoplastic transformation depending on cellular context [19].

In this study, we focus on the expression of CXCR2 and SOCS-3 in RCC. We chose to investigate CXCR2 and not CXCR1 because of experimental evidence underlining the importance of CXCR2/CXCR2 ligand in RCC biology [20], although the clinical relevance of this axis is unknown. Characterization of SOCS-3 expression, on the other hand, in tissue samples of RCC has not thus far been performed, despite its suspected involvement in the response of RCC to IFN-α by virtue of its interaction with JAK/STAT signaling, as alluded to [18]. First, we analyzed the relationships of CXCR2 with the proangiogenic cytokines and of SOCS-3 with p-STAT-3 in a series of RCC patients. Immunohistochemistry was validated by Western immunoblotting in 5 cases. Second, we examined the relationships of these molecules with VEGF and microvascular characteristics, aiming to gain insight into their potential involvement in the angiogenic process. Third, we tested the correlation of these molecules with p-JAK2 and the transcription factors p65/RelA (NFκB), p-c-Jun (AP-1), HIF-1a, and p53 by Western immunoblotting or immunohistochemistry in a subset of cases. Finally, we examined their potential impact on survival, progression and metastasis.

## Methods

### Patients

This is a study of 118 patients with RCC (diagnosed between 1996 and 2008) for whom archival primary tumor material at diagnosis, prior to chemotherapy, was available. In all cases, the histological diagnosis and grading were peer-reviewed by two pathologists (PK, AS) according to the principles laid down in the World Health Organization classification [21]. This study was approved by the University of Athens Medical School Ethics Committee and informed consent was obtained from each patient before study enrollment. The stage of each tumor was assigned following the guidelines from the 7th edition of TNM classification [22] and was known for 106 patients: 28 patients had stage I and 15 had stage II, 24 had stage III and 39 had stage IV disease. Follow-up information was available for 94 patients. The characteristics of patients enrolled in the present study are presented in Table 1*.*

**Table 1:** Clinicopathological characteristics of 118 patients enrolled in the present investigation

	**Number of patients (%)**
**Gender**	
Female	31 (26.3%)
Male	87 (73.8%)
**Histological type**	
Clear cell RCC	99 (83.9%)
Papillary RCC	9 (7.63%)
Chromophobe RCC	5 (4.24%)
Other*	5 (4.24%)
**Histological grade (Fuhrman)**	
I	6 (5.08%)
II	46 (38.98%)
III	48 (40.68%)
IV	18 (15.25%)
**Stage**	
I	28 (23.73%)
II	15 (12.71%)
III	24 (20.34%)
IV	39 (33.05%)
Not available	12 (10.17%)
**Lymph node metastasis**	
No	73 (61.8%)
Yes	20 (17%)
Not available	25 (21.2%)
**Total number of metastases**	
Absence	30 (25.42%)
1	25 (21.19%
2	24 (20.34%)
3	11 (9.32%)
4	4 (3.39%)
Not available	24 (20.24%)
**Disease progression**	
Absence	19 (16.10%)
Presence	42 (35.60%)
Not available	57 (48.30%)
**Follow-up**	
Alive/censored	43 (45.74%) Follow-up: 49 months (6-108.8 months)
Dead of disease	51 (54.26%) Follow-up: 30.37 months (1.77-117.43 months)
	**Median (range)**
**Age**	61 (30-80)

*Other histological types include collecting duct carcinoma (1 case), unclassified carcinoma (1 case), Chromophobe carcinoma with oncocytoma (1 case) and multilocular cystic carcinoma (2 cases).

### Immunohistochemical staining

Immunostaining was performed on paraffin-embedded 4 μm sections of formalin fixed tumor tissue using the two-step peroxidase conjugated polymer technique (DAKO Envision kit, DAKO, Carpinteria, CA). The primary antibodies used are listed in Table 2. In negative controls primary antibodies were substituted with non-immune serum.

**Table 2:** Characteristics of primary antibodies used in immunohistochemical analysis

**Protein**	**No of cases**	**Clone**	**Company**	**Catalog no.**	**Raised in**	**Positive controls**	**Antigen retrieval method**	**Dilution and incubation time for immunohistochemistry**
IL-8	118	Polyclonal	Invitrogen Corporation, Camarillo, CA	AHC 0881	Rabbit	Normal tonsillar tissue	pH 6 (low)	1:50, 18 h 4°C
IL-6	118	Polyclonal	Santa Cruz Biotechnology, Santa Cruz, CA	SC 1265	Goat	Normal tonsillar tissue	pH 6 (low)	1:50, 18 h 4°C
SOCS-3	118	Polyclonal	Santa Cruz Biotechnology, Santa Cruz, CA	SC 9023	Rabbit	Cholangiocarcinoma	pH 6 (low)	1:100, 18 h 4°C
CXCR2	118	Monoclonal	R&D Systems, Abingdon, England	MAB 331	Mouse	Normal tonsillar tissue	pH 9 (high)	1:100, 18 h 4°C
VEGF	117	Monoclonal	Pharmingen BD Company, San Diego, CA	clone G153-694	Mouse	Glioblastoma	pH 6 (low)	1:40, 18 h 4°C
p-STAT3 [specific at site Tyr 705]	117	Monoclonal	Cell Signaling Technology Inc., Boston, MA, USA	D3A7 XP	Rabbit	Human breast cancer	pH 6 (low)	1:100, 18 h 4°C
CD31	111	Monoclonal	DAKO	clone JC70A	Mouse	Kaposi sarcoma	pH 9 (high)	1:20, 18 h 4°C
P53	30	Monoclonal	DAKO	IR616	Mouse	Serous ovarian carcinoma	pH 6(low)	Pre-diluted, 1 h 37°C
HIF-1a	30	Monoclonal	Neomarkers Inc. Fremont CA, USA	MS 1164P	Mouse	Glioblastoma	pH 6(low)	Pre-diluted, 1 h 37°C
p65/RelA	30	Polyclonal	Zymed laboratories Inc., California, USA	18-7308	Rabbit	breast carcinoma	pH 6(low)	1:300, overnight 37°C

Evaluation of immunostained slides stained with IL-8, IL-6, SOCS3, CXCR2, p65/RelA, HIF-1a, p53 and VEGF was performed using light microscopy by two experienced pathologists (PK, AS) without knowledge of the clinical information and a Histo-score (H-score) based on the percentage of neoplastic cells displaying cytoplasmic immjunoreactivity multiplied by staining intensity was calculated. p65/RelA, HIF-1a and p53 were assessed in 30 random cases, whereas the remaining antibodies in the entire cohort. p-STAT3 nuclear staining and microvessel characteristics were evaluated using computerized image analysis software Image Pro software v5.1 (Media Cybernetics Inc.) on a Pentium III PC, as described previously [23]. The stained slides for CD31 and anti-pSTAT-3 were examined field by field at low magnification (×40 OLYMPUS BX51TF microscope) to identify the area showing the most intense vascularisation (i.e. the “hot spot”) and the highest H-score respectively. For CD31 the vascular hot spot area was photographed at ×200 magnification (OLYMPUS SC-30 Digital Camera) and stored as TIFF image file (2048 × 1532 pixels, RGB, 24-bit). For each countable microvessel several morphometric parameters were automatically established: major axis length (i.e. the distance between the two points along the vessel periphery that are further apart), minor axis length (i.e. the longest axis perpendicular to major axis formed by two points along the vessel periphery), perimeter, area (luminal plus endothelial cell area), Feret diameter ($\sqrt{\frac{4*\text{area}}{\pi }}$), shape factor ($\frac{4\pi *\text{area}}{\text{perimete}r^{2}}$), compactness ($\frac{\text{perimete}r^{2}}{\text{area}}$), MVD (microvessel density, i.e. the total count of microvessels per optical field) and TVA (total vascular area, i.e. the total area occupied by microvessels). For each case the mean value of major and minor axis length, area, perimeter, Feret diameter, shape factor and compactness along with MVD and TVA were recorded for statistical analysis. In cases where the most vascularized area was not obvious, two or more optical fields were photographed and the field with the highest MVD was finally chosen for further analysis.

### Western immunoblotting analysis

Western immunobloting analysis of IL-6, IL-8, CXCR2, SOCS-3, p-STAT-3, p-JAK2 and p-c-Jun expression was also performed on five RCC samples. After homogenization and fractionation of fresh frozen tumor tissue, 100 μg protein was separated on a 10% polyacrylamide gel and blotted onto nitrocellulose membranes, probed with primary antibody overnight, followed by incubation with horseradish peroxidase (HRP)-conjugated goat-anti-rabbit IgG or HRP-conjugated goat-anti-mouse IgG secondary antibody (AP132P and AP124P respectively, Chemicon, Millipore, Temecula, CA, USA). The same primary antibodies described in Table 2 were used at the following dilutions: 1:2,000 for anti–p-STAT-3, 1:200 for anti–SOCS-3, anti-CXCR2, and anti-IL-6. The anti-IL-8 antibody was diluted to a concentration of 0.1 μg/mL. The anti-p-JAK2 (sc-16566-R, Santa Cruz, 200 μg/ml) and anti-p-c-Jun (sc-822, Santa Cruz, 200 μg/ml) were diluted at 1:200. Bands were visualized using ECL chemiluminescence detection reagents (Perkin Elmer, Athens, Greece). Relative protein amounts were evaluated by a densitometric analysis using Image J software (La Jolla, CA, USA) and normalized to the corresponding Actin levels. All experiments have been performed at least 3 times and representative results of one experiment are shown.

### Statistical analysis

Statistical analysis was performed by a M.Sc. Biostatistician (GL). In the basic statistical analysis IL-6, IL-8, SOCS-3, CXCR2, VEGF, p65/RelA, HIF-1a, p53, p-STAT-3 expression and microvascular characteristics were treated as continuous variables. Associations with clinicopathological parameters and microvascular characteristics were tested using non-parametric tests with correction for multiple comparisons.

The set of microvascular parameters was subjected to factor analysis using the principal component extraction method. Three factors were extracted. The first factor represented microvessel caliber encompassing area, perimeter, Feret diameter and major and minor axis length. The second one represented microvessel shape (shape factor and compactness), whereas the third one represented the extent of microvascular network. The estimated factor scores were used in multivariate survival analysis.

Survival analysis was performed using death by disease as an endpoint. The effect of various clinicopathological parameters on clinical outcome was assessed by plotting survival curves according to the Kaplan-Meier method and comparing groups using the log-rank test. Numerical variables were categorized on the basis of cut-off values provided by ROC curves. Multivariate analysis was performed using stepwise forward Cox’s proportional hazard estimation model. Power estimation of the log-rank tests regarding SOCS3 and CXCR2 H-score was performed using the Freedman method for estimation of censored data. Statistical calculations were performed using the Statistical package STATA 11.0 for Windows. All results with a two-sided p level ≤0.05 were considered statistically significant, whereas a p-value between 0.05 and 0.10 was considered of borderline significance.

## Results

### Western blot analysis

**Figure 1:**
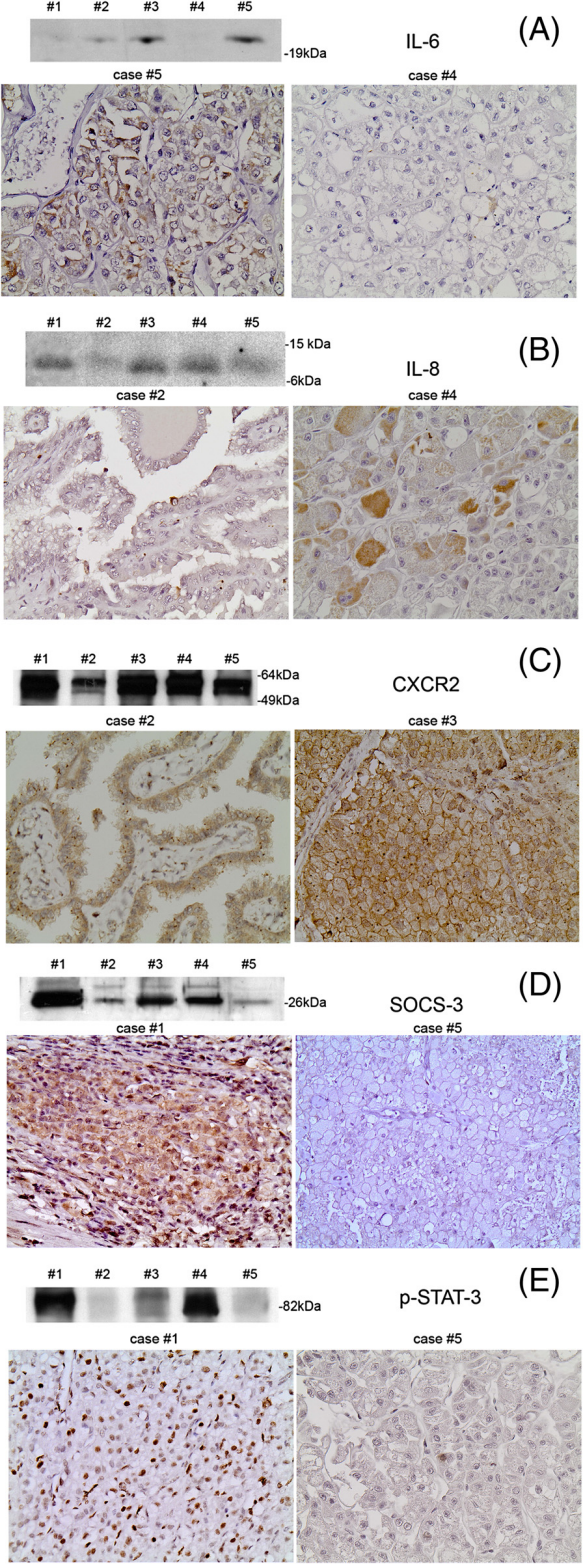
**IL-6, IL-8, CXCR2, SOCS-3 and p-STAT-3 protein levels in fresh-frozen tissue specimens by Western blot and immunohistochemical expression of CXCR2, SOCS-3 and IL-8 in formalin-fixed tissue from 2 representative cases (#1, 4: Clear cell RCCs, #2: Papillary RCC, #3, 5: Chromophobe RCCs).** Detection of IL-6 **(A),** IL-8 **(B)** CXCR2 **(C)** SOCS-3 **(D)** and p-STAT-3 **(E)** in the 5 cases. Western immunoblotting validated the results of immunohistochemistry.

The expression levels by Western blot in the examined 5 cases were found to correlate with the immunohistochemical expression of IL-6, IL-8, CXCR2, p-STAT-3 and SOCS-3 (Figure 1; Additional file 1: Figure S1-5).

### Immunohistochemical assessment of IL-6, IL-8 and CXCR2 expression in RCC and correlation with clinicopathological parameters

IL-6 and IL-8 expression were detected in 101/118 (85.6%) and 58/118 (49.15%) cases with the H-score ranging from 1-300 and 0.01-100 respectively (Figure 1A, B; Additional file 1: Figure S1, Additional file 2: Figure S2). CXCR2 was expressed in 112/118 cases with the H-score ranging from 2-285 (median value in positive cases: 80) (Figure 1C; Additional file 3: Figure S3). Immunoreactivity for all three antibodies was localized in the cytoplasm of neoplastic cells, increasing around necrosis. Endothelial cells and scattered macrophages displayed CXCR2 immunoreactivity which was also seen in the epithelial cells of distal proximal tubules and collecting ducts, albeit at lesser intensity.

IL-6 and IL-8 were coexpressed in 52/118 (44.06%) cases. Coexpression of IL-6 and CXCR2 was observed in 97/118 (82.2%) with only fifteen of CXCR2 positive cases being negative for IL-6 (15/112, 13.4%). Coexpression of IL-8 and CXCR2 was observed in 58/118 (49.15%) cases, with a significant number of CXCR2 positive cases (54/112 48.2%) being negative for IL-8. The vast majority, however, of these cases (45/54, 83.3%) expressed IL-6.

The correlations among the molecules under study are shown in Table 3. A significant positive correlation emerged between IL-6 and CXCR2 as well as between IL-8 and CXCR2. The former relationship, however, lost its statistical significance in a multivariate regression model including VEGF. Moreover, IL-6 H-score was marginally higher in the cases positive for IL-8.

**Table 3:** Correlations among IL-6, IL-8, CXCR-2, SOCS-3 and VEGF H-score in the entire cohort (Results of Spearman correlation coefficient)

	**IL-6**	**IL-8**	**CXCR2**	**SOCS-3**	**p-STAT-3**	**VEGF**
**IL-8**	NS*					
**CXCR2**	R = 0.2393, p = 0.0091***	R = 0.2989, p = 0.0010	NS			
**SOCS-3**	NS	NS	NS	NS		
**p-STAT3**	NS	NS	NS	NS	NS	
**VEGF**	R = 0.2623, p = 0.0054	R = 0.1217, p = 0.0730	R = 0.3549, p = 0.0001	R = 0.2540, p = 0.0071**	NS	NS

(NS: not significant).

*IL-6 H-score was marginally higher in the cases positive for IL-8 (Mann Whitney U test, p = 0.0992).

**This correlation failed to attain statistical significance in a multivariate model, including the presence of metastasis, to which both were significantly related.

***This relationship lost its statistical significance in a multivariate regression model including VEGF.

**Figure 2:**
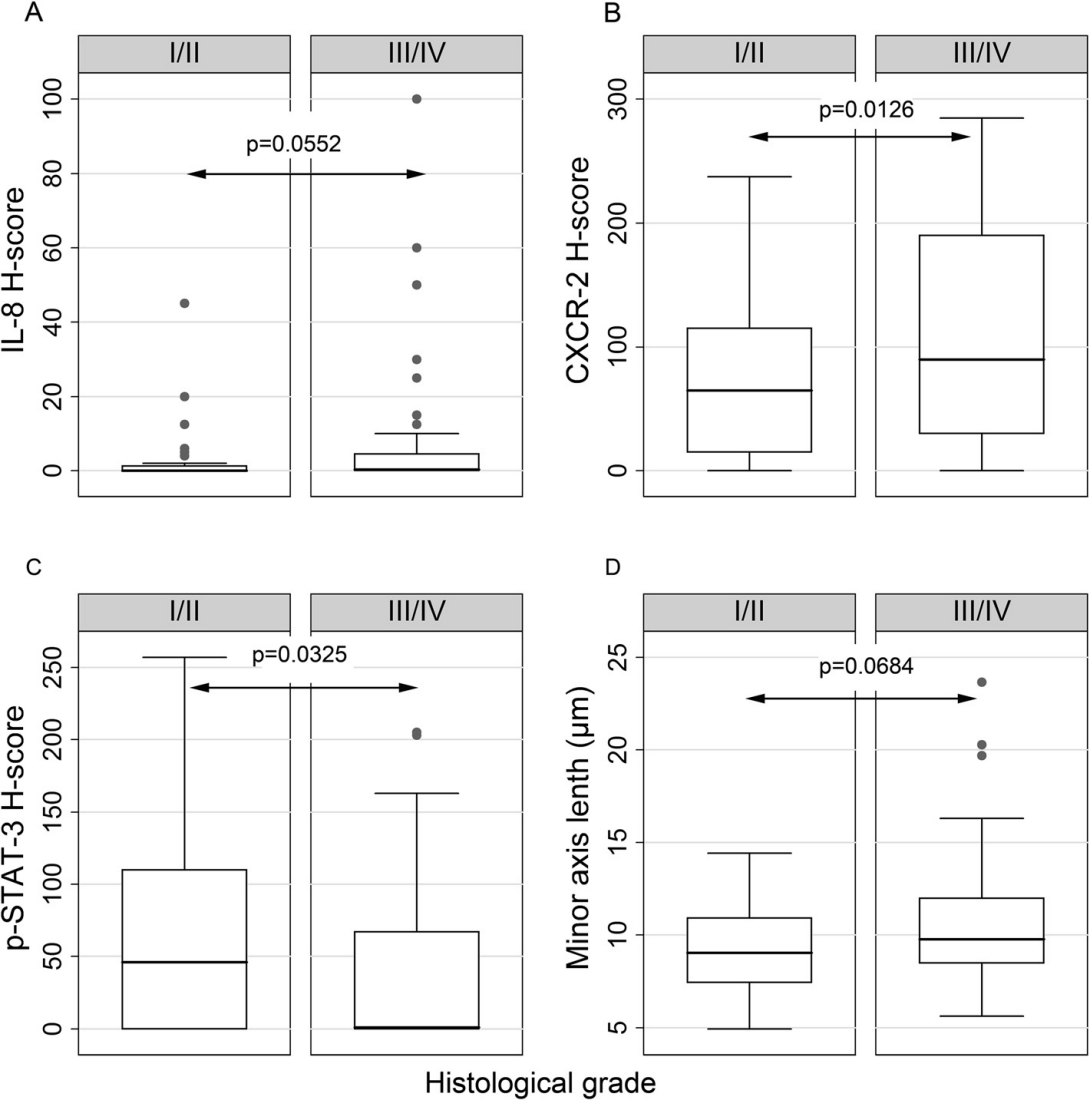
Box plots illustrating the correlations between IL-8 (A), CXCR-2 (B), p-STAT-3 (C) H-score and minor axis length (D) with histological grade.

**Figure 3:**
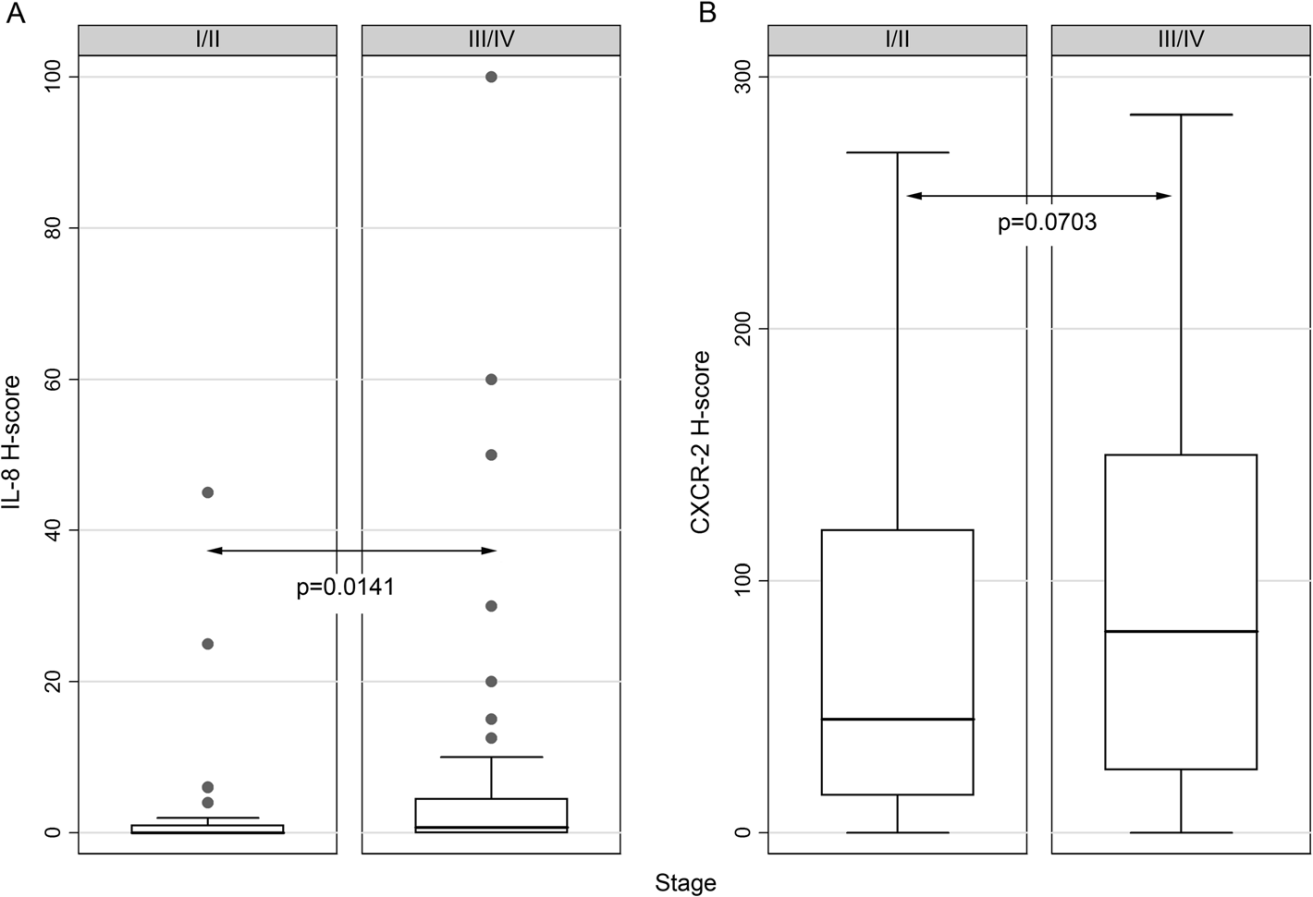
Box plots illustrating the correlations between IL-8 (A) and CXCR-2 (B) H-score with stage.

**Figure 4:**
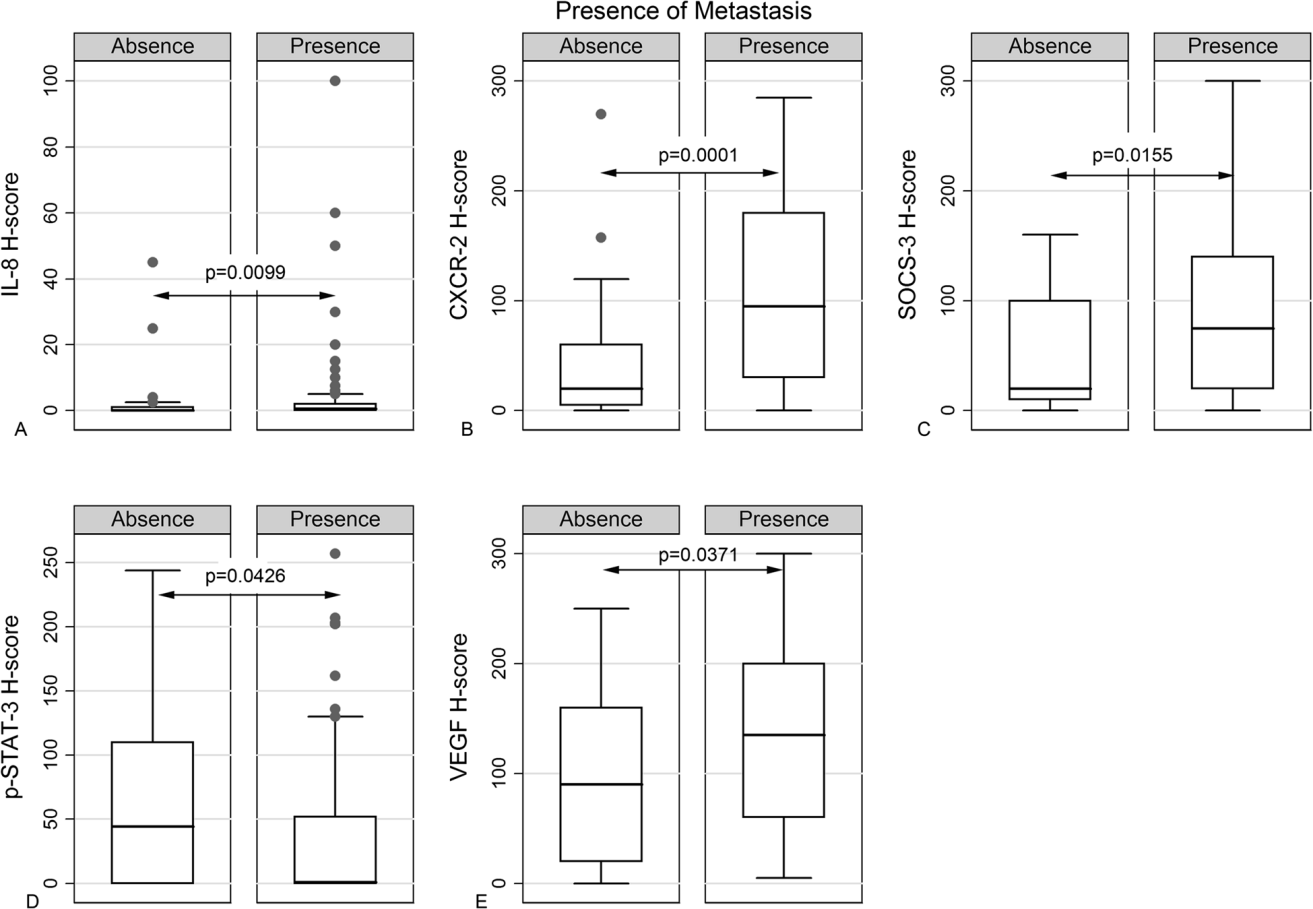
Box plots illustrating the correlations between IL-8 (A), CXCR-2 (B), SOCS-3 (C), p-STAT-3 (D) and VEGF (E) H-score with the presence of metastases.

The correlations between the molecules under study and clinicopathological characteristics are shown in Table 4. IL-8 expression levels were positively associated with histological grade and tumor stage (Figures 2A and 3A), the former relationship being of borderline significance. Accordingly, CXCR2 H-score increased in parallel with histological grade and marginally with tumor stage (Figure 2B and 3B). Interestingly, both IL-8 and CXCR2 H-scores were correlated with the presence (Figure 4A, B) and the total number of metastases. All other relationships of IL-6, IL-8 and CXCR2 H-score with clinicopathological features were not significant.

**Table 4:** Associations of the molecules under study with clinicopathological parameters (NS: not significant)

		**IL-6 H-score**		**IL-8 H-score**		**CXCR2 H-score**		**SOCS-3 H-score**		**p-STAT-3 H-score**		**VEGF H-score**	
	**n**	**Median (range)**	**p- value**	**Median (range)**	**p- value**	**Median (range)**	**p- value**	**Median (range)**	**p- value**	**Median (range)**	**p- value**	**Median (range)**	**p- value**
**Histological type**			NS		NS		NS		NS		NS		NS
Clear cell	99	20 (0-300)		0 (0-100)		80 (0-285)		60 (0-300)		4.5 (0-257)		127.5 (0-300)	
Papillary	9	30 (0-270)		0 (0-25)		140 (0-270)		75 (0-285)		24 (0-202)		120 (0-200)	
Chromophobe	5	135 (10-180)		1.5 (0-45)		15 (0-160)		22.5 (0-190)		1 (0-52)		100 (20-180)	
Other	5	60 (0-300)		1 (0-20)		20 (2-80)		22.5 (0-130)		0 (0-244)		120 (30-120)	
**Histological grade**			NS		0.0552		0.0126		NS		0.0325		NS
I/II	52	52.5(0-300)		0 (0-45)		65 (0-237.5)		38.75 (0-285)		46 (0-257)		127.5 (0-300)	
III/IV	66	15 (0-300)		0.35 (0-100)		90 (0-285)		67.5 (0-300)		1 (0-205)		120 (0-300)	
**Stage**			NS		0.0141		0.0703		NS		NS		NS
Ι/II	43	30 (0-300)		0(0-45)		45(0-270)		37.5 (0-285)		33 (0-244)		97.5 (1.5-300)	
ΙΙI/IV	63	20 (0-300)		0.75(0-100)		80 (0-285)		60 (0-300)		1 (0-257)		120 (0-300)	
**Presence of metastasis**			NS		0.0099		0.0001		0.0155		0.0426		0.0371
Absence	30	30 (0-300)		0 (0-45)		20 (0-270)		20 (0-160)		44.5 (0-244)		90 (0-250)	
Presence	64	17.5 (0-300)		0.5 (0-100)		95 (0-285)		75 (0-300)		1 (0-257)		135 (5-300)	
**Disease progression**			NS		NS		NS		0.0401		NS		NS
Absence	19	20 (0-200)		0.75 (0-10)		120 (16-270)		50 (0-180)		1 (0-257)		140 (10-300)	
Presence	42	12.5 (0-300)		0.75 (0-100)		90 (0-285)		95 (205-300)		10(0-207)		145 (5-300)	
**Total number of metastases**	94	R = 0.3458, p = 0.0006		R = 0.3333, p = 0.0010		NS		R = 0.2945, p = 0.0040		R = -0.2212, p = 0.0331		NS	
**Patients’ age**	118	R = 0.2228, p = 0.0871		NS		NS		NS		NS		NS	

### Immunohistochemical assessment of SOCS-3 and p-STAT-3 expression in RCC and correlation with clinicopathological parameters

SOCS-3 expression was cytoplasmic or membranous and was detected in 111/118 (94.07%) cases (Figure 1D; Additional file 4: Figure S4). p-STAT-3 expression was nuclear and was recorded in 84/117 (71.79%) (Figure 1E; Additional file 5: Figure S5). Endothelial and inflammatory cells were also positive for SOCS3 and p-STAT-3 and therefore served as internal positive controls. Weak SOCS-3 expression was noted in distal tubules and collecting ducts. Coexpression of SOCS-3 and p-STAT-3 was observed in 79/117 (67.52%) cases. SOCS-3 H-score was marginally higher in the cases positive for p-STAT-3 (p = 0.0707).

SOCS-3 H-score was positively correlated with the presence (Figure 4C) and the total number of metastases, as well as with disease progression. An inverse correlation between p-STAT-3 H-score and histological grade (Figure 2C), the presence (Figure 4D) and the total number of metastases was also established.

### Relationship of IL-6, IL-8, SOCS-3, CXCR2 and p-STAT-3 expression with VEGF

VEGF H-score was positively correlated with IL-6, CXCR2 and IL-8, the latter relationship being of marginal significance (Table 3). Interestingly, although SOCS-3 and VEGF seemed to be positively correlated, when we adjusted a multivariate model including VEGF and SOCS-3 H-score along with the presence of metastasis, a parameter with which both molecules were significantly correlated, the respective relationship between VEGF and SOCS-3 failed to attain statistical significance (Table 3).

### Relationship of VEGF, IL-6, IL-8, SOCS-3, CXCR2 and p-STAT3 expression with microvascular characteristics

The correlations among IL-6, IL-8, CXCR-2, SOCS-3 and VEGF H-score andmicrovascular characteristics are shown in Table 5. Significant positive correlations emerged between VEGF H-score and microvessel area, TVA or Feret diameter. Moreover, CXCR2 was inversely correlated with major axis length, perimeter, area, minor axis length, Feret diameter and compactness, the latter four correlations being of marginal significance, whereas it was positively correlated with shape factor. Furthermore, SOCS-3 H-score increased in parallel with shape factor and was inversely correlated with compactness. Although, IL-8 seemed to be negatively correlated with MVD, when we adjusted a multivariate model including MVD and IL-8 H-score along with histological grade, the respective relationship between these two molecules failed to attain statistical significance.

**Table 5:** Correlations between IL-6, IL-8, CXCR-2, SOCS-3 and VEGF H-score and microvascular characteristics (Results of Spearman correlation coefficient)

	**MVD**	**TVA**	**Major axis length**	**Minor axis length**	**Area**	**Perimeter**	**Feret diameter**	**Compactness**	**Shape factor**
IL-6	NS	NS	NS	NS	NS	NS	NS	NS	NS
IL-8	R = -0.1873, p = 0.0432**	NS	NS	NS	NS	NS	NS	NS	NS
CXCR2	NS	NS	R = -0.1884, p = 0.0419	R = -0.1804, p = 0.0516	R = -0.1541, p = 0.0972	R = -0.1818, p = 0.0497	R = -0.1541, p = 0.0972	R = -0.1756, p = 0.0582	R = 0.1758, p = 0.0579
SOCS-3	NS	NS	NS	NS	NS	NS	NS	R = -0.1686, p = 0.0693	R = 0.1684, p = 0.0695*
pSTAT3	NS	NS	NS	NS	NS	NS	NS	NS	NS
VEGF	NS	R = 0.2166, p = 0.0230	NS	NS	R = 0.1967, p = 0.0394	NS	R = 0.1967, p = 0.0394	NS	NS

NS: not significant.

*When analysis was performed on a categorical basis SOCS-3 H-score was significantly correlated with shape factor (Mann Whitney U test, p = 0.0286).

**This relationship failed to attain statistical significance in a multivariate model including MVD and IL-8 H-score along with histological grade, the latter emerging as a confounder in the correlation between these two molecules.

### Relationship of VEGF and microvascular characteristics with clinicopathological features

**Figure 5:**
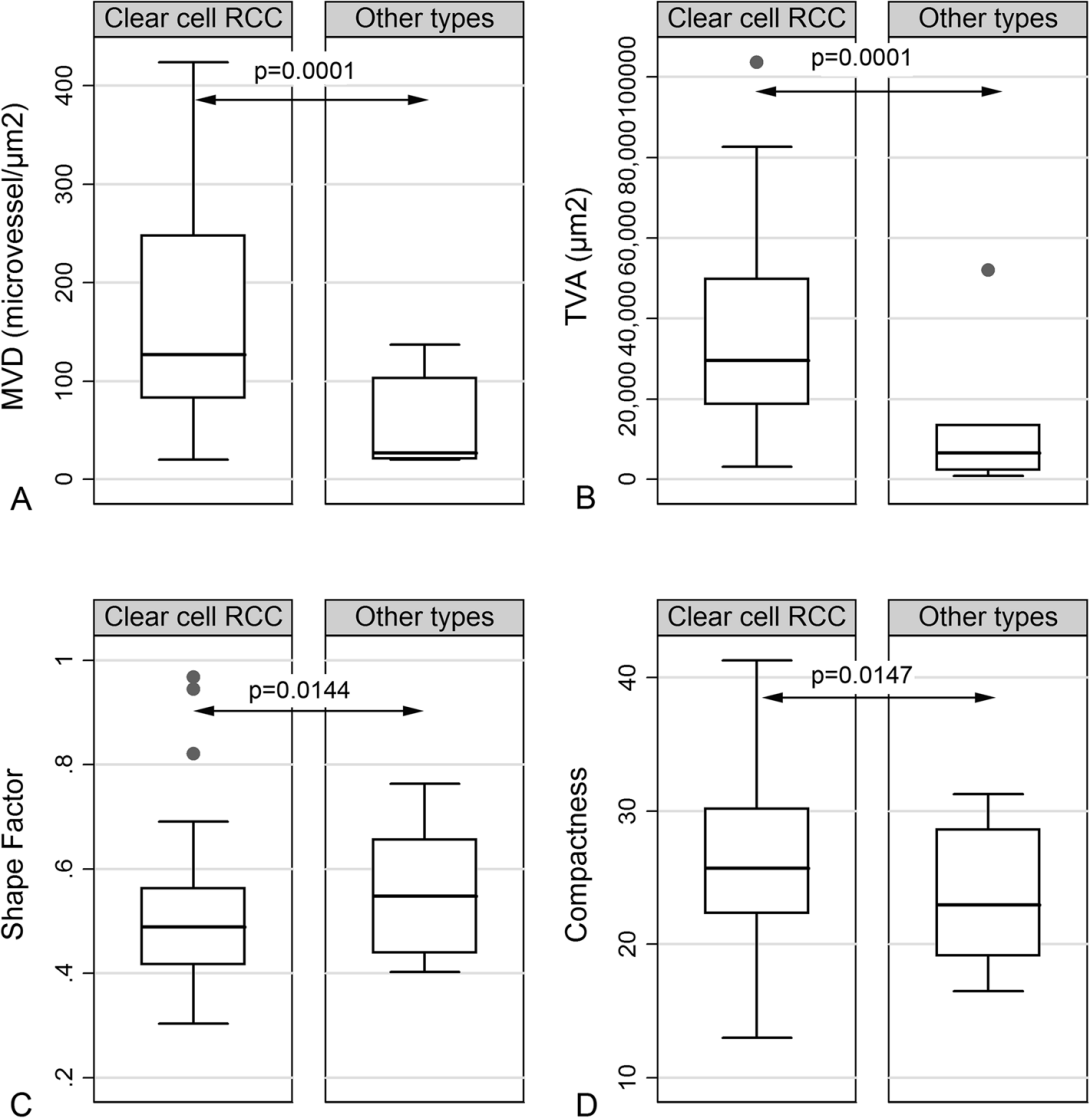
Box plots illustrating the correlations between MVD (A), TVA (B), shape factor (C) and compactness (D) with histological type.

**Figure 6:**
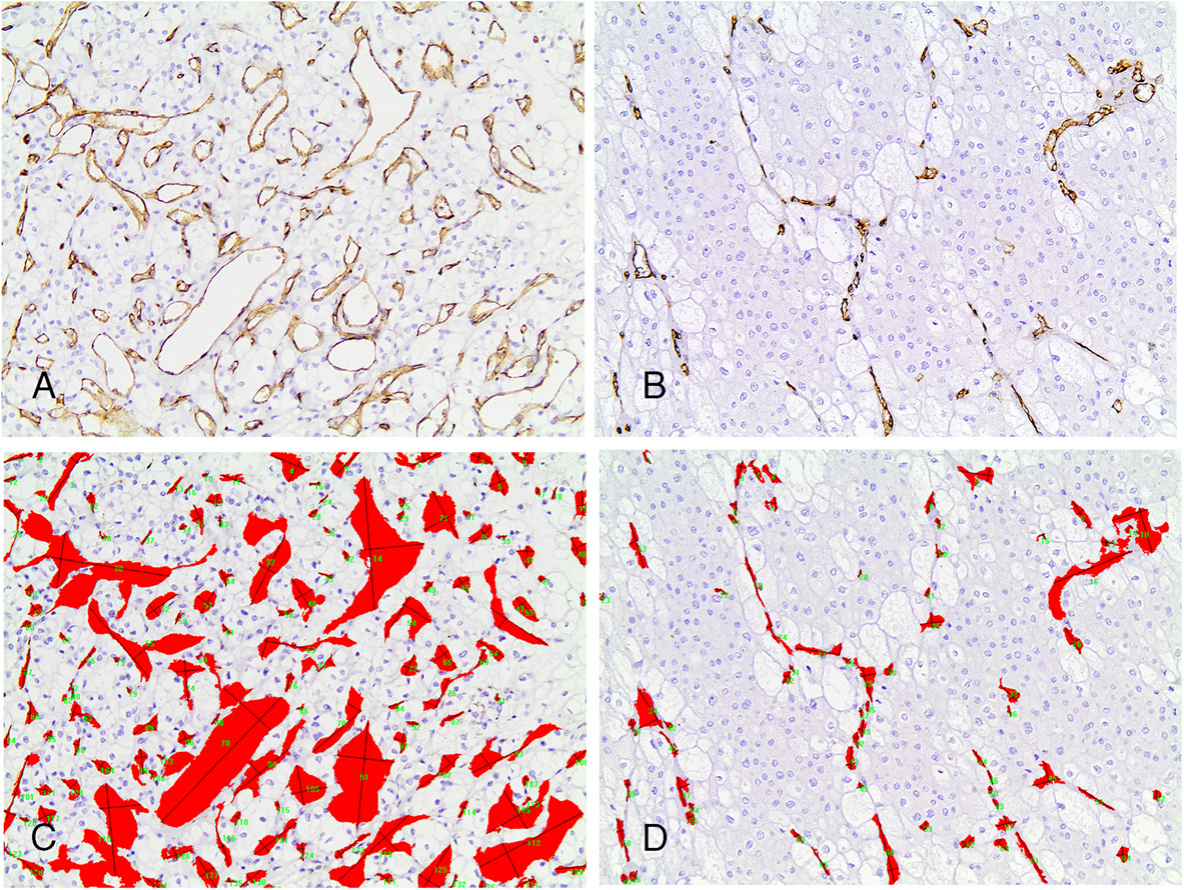
**Immunohistochemical staining of a clear cell (A) and a chromophobe (B) RCC.(C, D)** Same fields as in **(A, B)**. The outline of each vessel is traced; the red layer represents the section area of each vessel. Clear cell RCC **(A, C)** displays clearly higher MVD and TVA when compared to chromophobe RCC **(B, D)**.

VEGF was correlated with the presence of metastasis (Figure 4E). Moreover, minor axis length was positively correlated with histological grade (p = 0.0684, Figure 2D), being marginally higher in grades III/IV. As expected, clear cell carcinomas displayed higher MVD and TVA as well as compactness (p = 0.0001 for MVD and TVA, and p = 0.0147 for compactness) and lower levels of shape factor (p = 0.0144) when compared to the remaining histological types (Figures 5A-D and 6), consistent with the much higher vascularity of clear cell RCC, as compared to the remaining types [1,2]. The increased compactness and lower shape factor values of microvessels in clear cell RCC illustrate the presence of collapsed vessels sections indicative of decreased intraluminal pressure (according to Bernoulli’s law) and consequently enhanced intratumoral blood flow [23].

### Relationship of IL-6, IL-8, SOCS-3, CXCR2 with p65/RelA, HIF-1a, p53, p-JAK2 kinase and p-c-Jun

**Figure 7:**
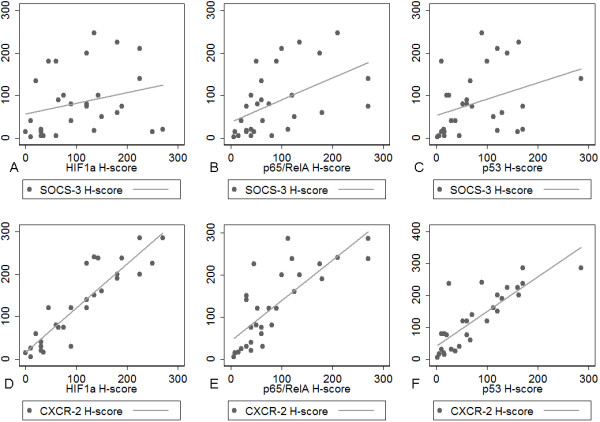
**Plots illustrating the correlations of SOCS-3 and CXCR2 with p65/RelA, HIF-1a and p53 in the 30 cases analyzed.** (A, B, C) SOCS-3 with p65/RelA, HIF-1a and p53. (D, E, F) CXCR2 with p65/RelA, HIF-1a and p53.

SOCS-3 and CXCR2 were positively correlated with HIF-1a (R = 0.3675, p = 0.0498 for SOCS-3 Figure 7A and R = 0.9050, p < 0.0001 for CXCR2, Figure 7D), p65/RelA (R = 0.6204, p = 0.0003 for SOCS-3 Figure 7B and R = 0.8069, p < 0.0001 for CXCR2, Figure 7E) and p53 (R = 0.4303, p = 0.0198 for SOCS-3, Figure 7C and R = 0.8254, p < 0.0001 for CXCR2, Figure 7F) H-score. The correlations between IL-6 or IL-8 with p65/RelA, HIF-1a, p53 were not significant (p > 0.10).

**Figure 8:**
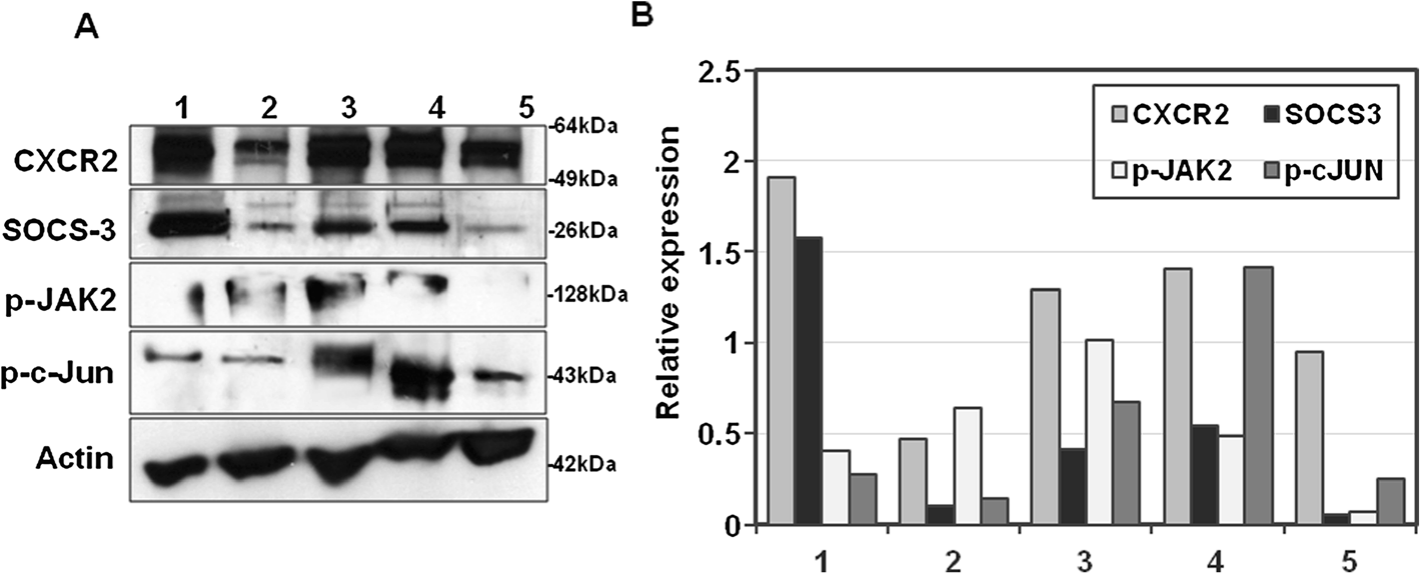
**Expression of CXCR2, SOCS-3, p-JAK-2 and p-c-Jun in 5 cases.** Western immunoblotting of CXCR2, SOCS-3, p-JAK2 and p-c-Jun expression in 5 tumor cases **(A)**. Densitometric analysis of relative protein amounts normalized to the corresponding Actin levels was performed using Image J software **(B)**.

Furthermore, Western blot analysis of 5 RCC cases revealed that increased expression of SOCS-3 was associated with decreased p-JAK2 and p-c-Jun expression and vice versa (Figure 8).

### Survival analysis

**Figure 9:**
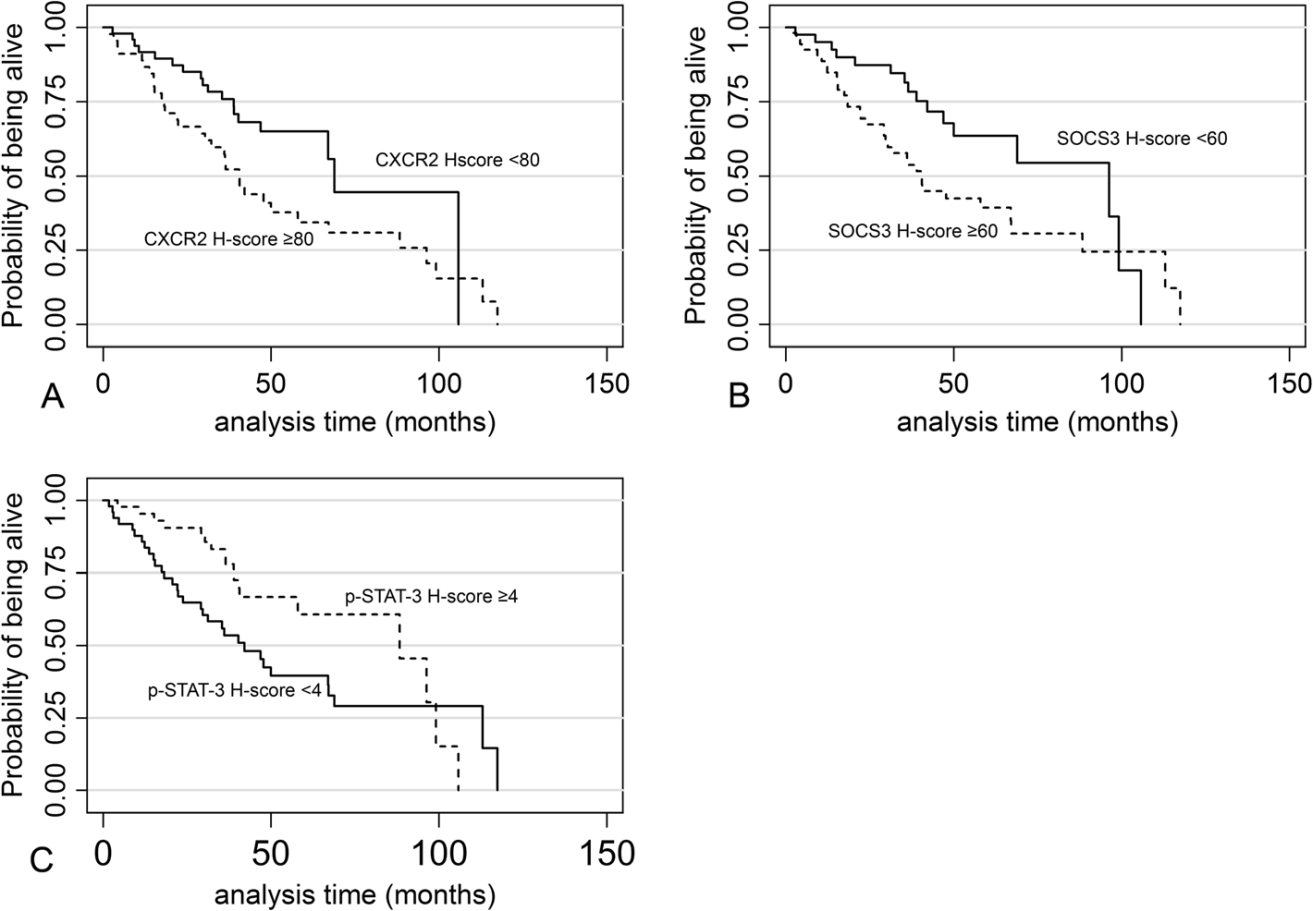
Kaplan-Meier curves for cancer specific survival according to CXCR-2 (A), SOCS-3 (B) and p-STAT-3 (C) immunoexpression.

The results of univariate survival analysis are presented in Table 6. The parameters adversely affecting survival were advanced stage, increased CXCR2 (Figure 9A) and SOCS-3 (Figure 9B) and decreased p-STAT-3 (Figure 9C) H-score although the latter relationship was of marginal significance. The comparison of survival functions among the groups allocated by CXCR2 and SOCS3 H-score had a statistical power of 0.84 and 0.96 respectively at a significance level of 0.05.

**Table 6:** Results of Univariate survival analysis (log-rank test)

**Variables**	**Log-rank test (p-value)**
**Age**	
*<61 vs ≥61 years*	0.1142
**Gender**	
*Male vs Female*	0.5701
**Histological type**	
*Clear cell vs non- clear cell*	0.2441
**Histological grade**	
*I/ II vs III/IV*	0.2378
**Stage**	
*I/II vs III/IV*	**0.0002**
**Total number of metastases**	
*1 vs more*	0.2787
**IL-6 H-score**	
*<24 vs ≥24*	0.8718
**IL-8 H-score**	
*Negative vs positive*	0.1311
**CXCR2 H-score**	
*<80 vs ≥80*	**0.0293**
**SOCS3 H-score**	
<60 vs *≥60*	**0.0478**
**p-STAT3 H-score**	
<4 vs *≥4*	**0.0730**
**VEGF H-score**	
<120 vs *≥120*	0.8804
**MVD**	
<125 vs *≥125*	0.9391
**TVA**	
<30069.62 vs *≥*30069.62	0.2134
**Area**	
*<222.345 vs ≥222.345*	0.1057
**Major axis length**	
*<24.771 vs ≥24.771*	0.4925
**Minor axis length**	
*<9.541 vs ≥9.541*	0.3642
**Perimeter**	
*<74.329 vs ≥74.329*	0.1194
**Shape factor**	
*< 0.495 vs ≥0.495*	0.3118
**Feret diameter**	
*<16.826 vs ≥16.826*	0.1057
**Compactness**	
*<25.361 vs ≥25.361*	0.2241

Bold signifies a statistically significant result.

The results of multivariate survival analysis are presented in Table 7. SOCS-3 H-score emerged as an independent predictor of adverse prognosis, along with tumor stage.

**Table 7:** Cox proportional Hazards model with stepwise forward selection for the 94 patients with RCC

	**HR**	**P value**	**95% confidence interval of HR**	
SOCS3 H-score	1,004	0,045	1,000	1,008
Tumour stage	1,734	<0.001	1,277	2,355

IL-8, IL-6, CXCR2, SOCS-3, p-STAT3, VEGF, Microvascular factors and tumor stage were introduced into the model.

## Discussion

The angiogenic properties of Th2 cytokines (IL-6 and IL-8) have led to the inevitable conclusion that they may potentiate RCC growth, metastasis and immune evasion [2,24]. Despite experimental evidence implicating IL-8/CXCR2 axis and SOCS-3 in the progression of RCC, in situ characterization of their expression by RCC cells and its clinical relevance has not thus far been performed.

We herein describe for the first time the cytoplasmic immunolocalization of CXCR2 in neoplastic cells in the vast majority of our RCC cases, along with IL-6 and IL-8, disputing previously reported findings in a small series [20]. Our findings concur with the reported identification of CXCR2 mRNA and protein in the RCC cell line A-498 and in short term primary RCC cell cultures [25]. It is conceivable that such a widespread CXCR2 expression by the neoplastic cells could be attributed to HIF-1a, which is known to be constitutively active in RCC driving the acquisition of a hypoxic phenotype or to other hypoxia –inducible transcription factors [26,27]. To address this issue, we investigated the relationship between CXCR2 and HIF-1a, p53 or p65/RelA expression in a subset of RCC specimens. We verified that CXCR2 (but not IL-8) and these transcription factors are strongly interrelated, although the underlying mechanisms remain to be deciphered. For example, computational analysis has identified potential binding sites for HIF-1a and NF-κB in CXCR2 promoter in hypoxic prostate cancer cells [28] and p53 reportedly upregulates CXCR2 transcription by binding to CXCR2 promoter [29]. The increased Western blot levels of p-c-Jun we observed in RCC are also consistent with the reported decreased levels of c-Jun N-terminal kinase in CXCR2 knock-out mice [30]. The observed relationship between CXCR2 and VEGF in our series probably reflects the fact that they are both transcriptional targets of HIF-1a [27,28].

We also documented a liaison between CXCR2 and IL-8. Interestingly, 83.3% of CXCR2 positive/IL-8 negative cases exhibited IL-6 immunoreactivity, implying a redundancy of the angiogenic mechanisms in this tumor [31]. Both IL-8 and CXCR2 expression increased along with Fuhrman’s grade and stage advocating that IL-8/CXCR2 autocrine signaling underpins both the development and the progression of RCC and represents a mechanism adopted by diverse tumor types to augment their angiogenic, growth and metastatic potential [32]. On the contrary, IL-6 showed no association with grade or stage in our series.

A major finding is that IL-8/CXCR2 signaling may be implicated in the metastatic process of RCC, since their expression levels were correlated with the presence and/or number of metastases in our series. Several lines of *in vitro* and *in vivo* evidence corroborate this notion. First, IL-8 levels positively correlated with matrix metalloproteinases, which facilitate the metastatic process by degrading basement membranes rev. in ref [1]. Second, IL-8 tissue levels are reportedly higher in metastatic RCC [1]. Third, increased levels of CXCR2 have been recorded in endothelial cells of metastatic RCC [20]. Fourth, orthotopic RCC tumors displayed a reduced growth and metastatic potential in CXCR2 -/- mice [20].

It is of interest that CXCR2, but not IL-8 was associated with the presence of small caliber microvessels as well as with high values of shape factor corresponding to the presence of rounder vessel sections. This particular pattern of microvessels denotes an increase in intraluminal pressure because of retarded intratumoral blood flow within an abnormal vascular network [33].

Although CXCR2 expression was predictive of poor patient survival in univariate analysis, this effect did not hold true in multivariate analysis. Interestingly, IL-8 was not prognostically informative obviously indicating that CXCR2, representing the point of convergence of all ELR + chemokines may provide a more accurate estimate of tumors’ angiogenic or invasive potential than any individual chemokine upstream [34]. In this context, blockade or silencing of CXCR2 gene attenuated human pancreatic tumor growth [35] and arrested ovarian carcinoma cells at G_0_/G_1_ and G_2_/M [36]. Furthermore, CXCR2 has been shown to suppress the expression of proapoptotic factors while enhancing the expression of anti-apoptotic proteins [35], thereby assisting neoplastic cells to resist chemotherapy.

An intriguing observation in our study is that p-STAT-3 inversely correlated with grade, the presence and number of metastases and marginally with survival. This apparently disagrees with experimental studies in which transfection of dominant-negative STAT-3 completely abolishes the anti-apoptotic effect of IL-6 on RCC cells [37], but also with an early report implicating the constitutive activation of STAT-3 in the metastatic potential of RCC cells in a small series [38]. Such conflicting data reflects the dual role of STAT-3 harboring both tumor suppressive and oncogenic properties [23,39].

To the best of our knowledge, our study is the first to deal with the expression of SOCS-3 in RCC tissue samples. Given that SOCS-3 is a negative regulator of STAT-3 activation, it was initially believed that it might function as a tumor suppressor and, hence, its expression might be repressed in neoplasms, particularly those with constitutive activation of STAT-3. Our findings, however, fail to confirm this assumption, documenting SOCS-3 expression in the vast majority of RCC cases mostly accompanied by p-STAT-3 expression. We also failed to establish the expected negative correlation between SOCS-3 and p-STAT-3 in line with observations in other tumors [40,41]. The simultaneous presence of the two molecules in most RCC specimens is consistent with the notion that SOCS-3 is a transcriptional target of STAT-3 [19]. Alternatively, it could be hypothesized that neoplastic cells have developed strategies to by-pass negative regulation by SOCS-3 [40]. However, increased SOCS-3 levels were accompanied by decreased p-JAK-2 in Western blotting providing evidence for the operation of the negative feedback loop between SOCS-3 and JAK-2 signaling in RCC. Furthermore, increased SOCS-3 expression was found to correlate with reduced phosphorylation of c-Jun thus suggesting a possible suppression of AP-1 activity in RCC. In agreement with our finding endogenous SOCS-3 has been reported to block c-Jun phosphorylation and inhibit AP-1 activity in neuroblastoma cells [42]. Furthermore, we were able to elicit strong positive correlations between SOCS-3 and HIF-1a, p65/RelA and p53 in RCC recapitulating recently published evidence that HIF-1a functions as an important regulator of SOCS-3 in glioma cells [43] and that SOCS-3 overexpression enhances p53 phosphorylation in pleural mesothelioma cells by inhibiting its degradation [44].

One of the most important findings of the present investigation is the association of SOCS-3 expression with the presence and number of metastases, progression and diminished survival in RCC patients. It should be stressed that the adverse prognostic significance of SOCS-3 was maintained in multivariate survival analysis in the presence of stage, IL-6, IL-8, CXCR2, VEGF and microvascular factor scores. Taking into account the low expression of SOCS-3 in normal kidney, these findings bring forward SOCS-3 as a tumor promoter in RCC, endowing neoplastic cells with a survival advantage. In harmony with this assumption, SOCS-3 expression has been shown to increase during development and progression of prostate cancer [45] and enhances glioblastoma cell survival, its loss converting the anti-apoptotic function of STAT-3 into pro-apoptotic [46]. A recent study has augmented interest in SOCS-3 implicating it in the resistance to IFN treatment in RCC [47]. Thus, overexpression of SOCS-3 via gene transfection in IFN-sensitive RCC cells significantly diminished the growth inhibitory effect of IFN-α, Suppression of SOCS-3 by siRNA restored sensitivity in IFN-α resistant RCC cells and suppressed the growth of IFN-α resistant RCC xenograft [47], as well as of 786-O RCC cell line following the combined administration of anti-IL-6R and IFN-α [48].

## Conclusions

In summary, this is the first study highlighting the importance of SOCS-3 overexpression into RCC progression, metastatic process and biologic aggressiveness. More importantly, our data stands in favor of SOCS-3 as an independent prognostic marker and lays the ground for its therapeutic targeting in combination with IFN-α. IL-8/CXCR2 autocrine signaling also contributes to the angiogenic and metastatic phenotype of RCC cells, but may be of lesser importance as a therapeutic tool, although its targeting might augment the therapeutic benefit gained from SOCS-3 modulation and IFN-α treatment. Both CXCR2 and SOCS-3 appear to elaborate relationships with several transcription factors induced under hypoxia, such as HIF-1a, NF-κΒ, p53 and p-c-Jun. These findings should await validation in prospective studies enrolling a larger number of patients and allowing for subgroup analysis.

## Competing interests

The authors declare that they have no competing interests.

## Authors’ contributions

AS: evaluation of the immunohistochemical slides, editing part of the manuscript. GL: statistical analysis, editing part of the manuscript. C. Piperi: designing, performing and assessing Western blot analysis. CA: performing Western blot experiments. GD: performing Western blot experiments and quantitation diagrams. AB: organizing the collection of the entire patient series and participated in the collection of clinicolaboratory data. AK collecting clinical data of the patients. GAL: collecting data concerning patient’s follow-up. SP: providing fresh tissue samples in order to perform Western blot analysis. KS: providing fresh tissue samples in order to perform Western blot analysis. MAD: organizing the collection of the entire patient series. EP: contributing to the revision of the research project. HG: contributing to the revision of the manuscript. PK: supervising the whole research project, contributing to the writing of the manuscript, evaluating the immunohistochemical slides. All authors read and approved the final manuscript.

## Pre-publication history

The pre-publication history for this paper can be accessed here:

http://www.biomedcentral.com/1471-2407/14/149/prepub

## Supplementary Material

Additional file 1 **IL-6 protein levels in fresh-frozen tissue specimens by Western blot and immunohistochemical expression of IL-6 in formalin-fixed tissue in the same five cases (#1, 4: Clear cell RCCs, #2: Papillary RCC, #3, 5: Chromophobe RCCs).** Western immunoblotting validated the results of immunohistochemistry. Click here for file

Additional file 2 **IL-8 protein levels in fresh-frozen tissue specimens by Western blot and immunohistochemical expression of IL-8 in formalin-fixed tissue in the same five cases (#1, 4: Clear cell RCCs, #2: Papillary RCC, #3, 5: Chromophobe RCCs).** Western immunoblotting validated the results of immunohistochemistry. Click here for file

Additional file 3 **CXCR2 protein levels in fresh-frozen tissue specimens by Western blot and immunohistochemical expression of CXCR2 in formalin-fixed tissue in the same five cases (#1, 4: Clear cell RCCs, #2: Papillary RCC, #3, 5: Chromophobe RCCs).** Western immunoblotting validated the results of immunohistochemistry. Click here for file

Additional file 4 **SOCS-3 protein levels in fresh-frozen tissue specimens by Western blot and immunohistochemical expression of SOCS-3 in formalin-fixed tissue in the same five cases (#1, 4: Clear cell RCCs, #2: Papillary RCC, #3, 5: Chromophobe RCCs).** Western immunoblotting validated the results of immunohistochemistry. Click here for file

Additional file 5 **p-STAT-3 protein levels in fresh-frozen tissue specimens by Western blot and immunohistochemical expression of p-STAT-3 in formalin-fixed tissue in the same five cases (#1, 4: Clear cell RCCs, #2: Papillary RCC, #3, 5: Chromophobe RCCs).** Western immunoblotting validated the results of immunohistochemistry. Click here for file
