# Supplementary figures and images for: The role of CXC-chemokine receptor CXCR2 and suppressor of cytokine signaling-3 (SOCS-3) in renal cell carcinoma
Source: BMC Cancer. 2014 Mar 4;14:149. doi: 10.1186/1471-2407-14-149 (PMC4015755; doi:10.1186/1471-2407-14-149)

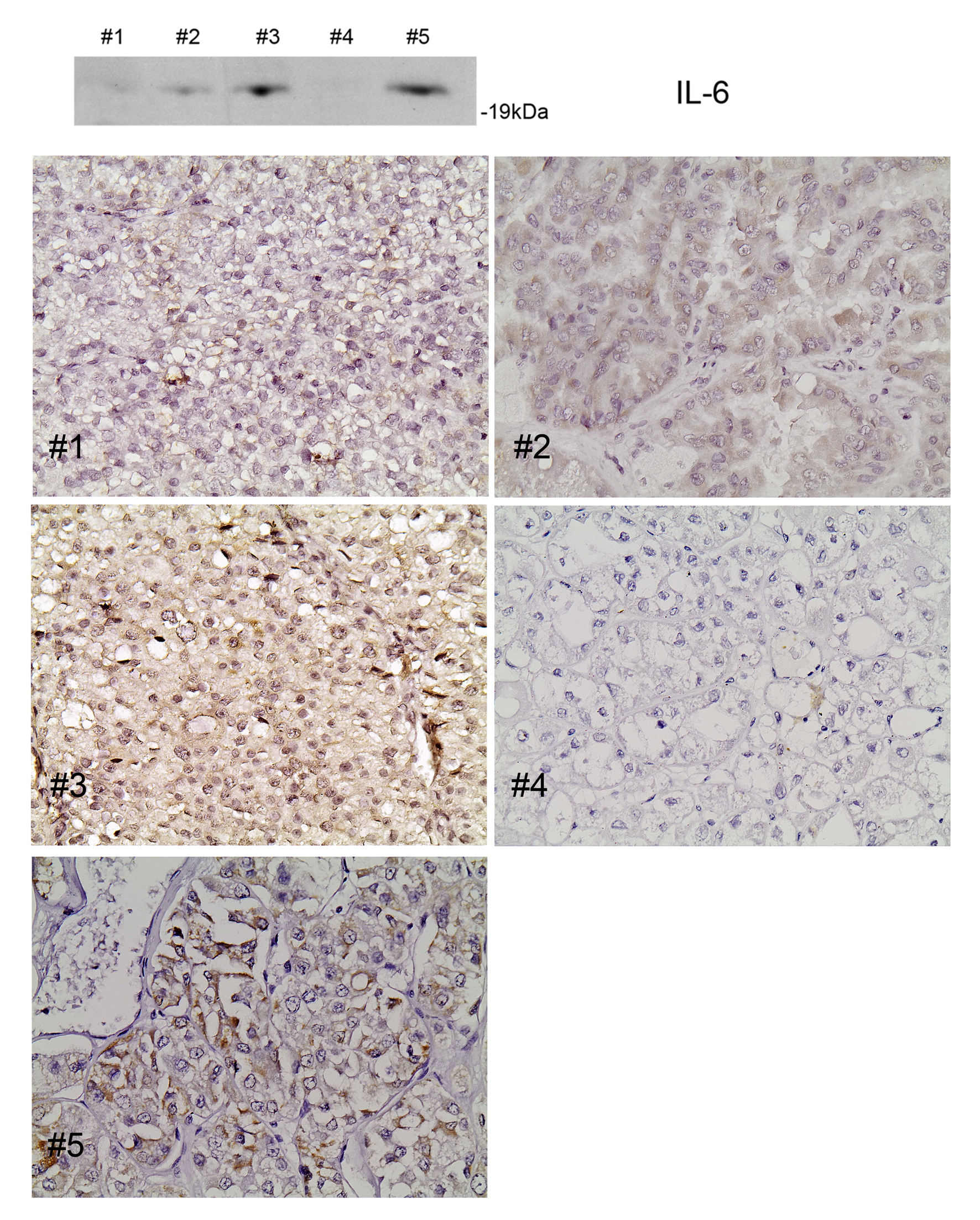

Supplement: Additional file 1 — IL-6 protein levels in fresh-frozen tissue specimens by Western blot and immunohistochemical expression of IL-6 in formalin-fixed tissue in the same five cases (#1, 4: Clear cell RCCs, #2: Papillary RCC, #3, 5: Chromophobe RCCs). Western immunoblotting validated the results of immunohistochemistry. [file 1471-2407-14-149-S1.tiff]

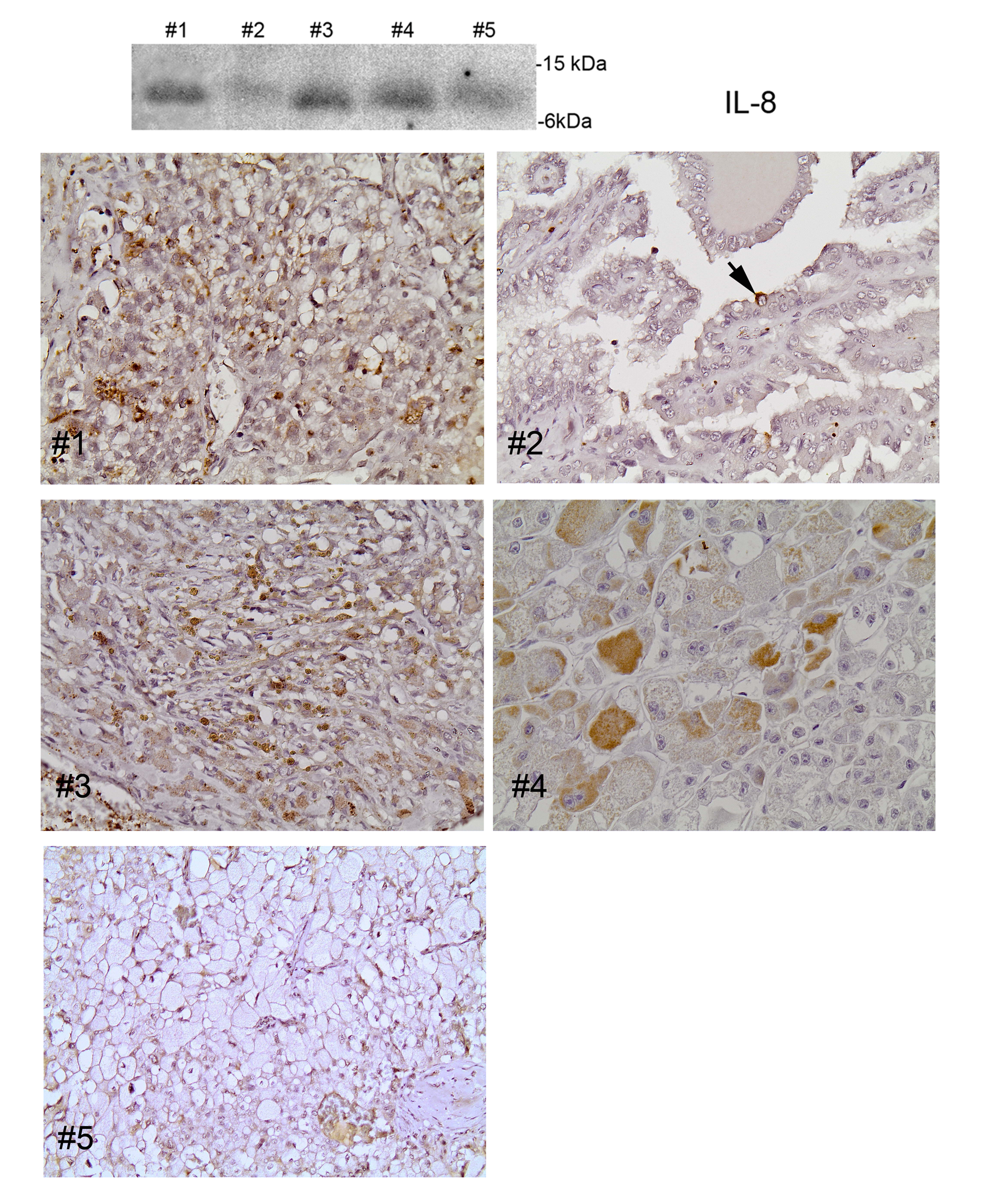

Supplement: Additional file 2 — IL-8 protein levels in fresh-frozen tissue specimens by Western blot and immunohistochemical expression of IL-8 in formalin-fixed tissue in the same five cases (#1, 4: Clear cell RCCs, #2: Papillary RCC, #3, 5: Chromophobe RCCs). Western immunoblotting validated the results of immunohistochemistry. [file 1471-2407-14-149-S2.tiff]

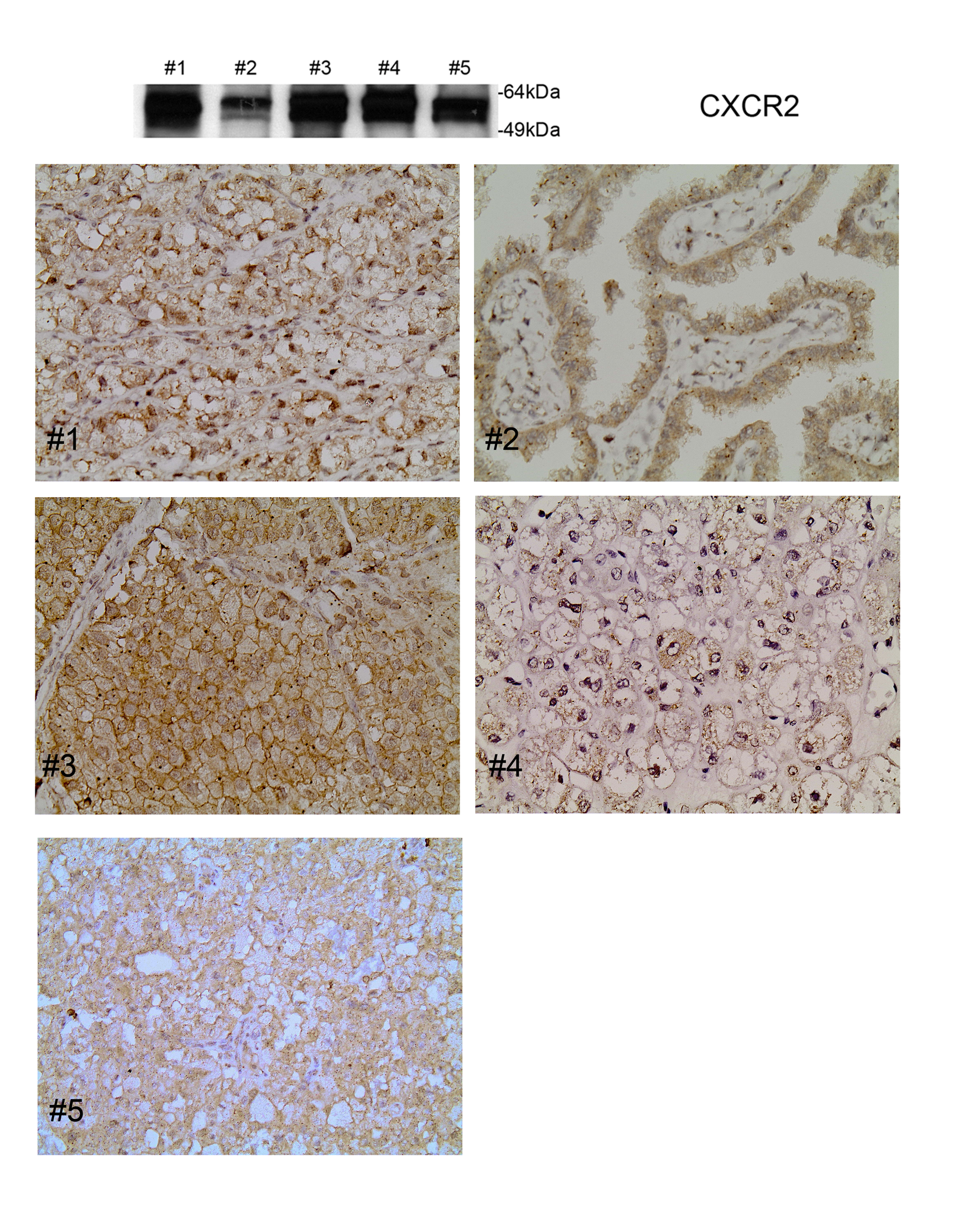

Supplement: Additional file 3 — CXCR2 protein levels in fresh-frozen tissue specimens by Western blot and immunohistochemical expression of CXCR2 in formalin-fixed tissue in the same five cases (#1, 4: Clear cell RCCs, #2: Papillary RCC, #3, 5: Chromophobe RCCs). Western immunoblotting validated the results of immunohistochemistry. [file 1471-2407-14-149-S3.tiff]

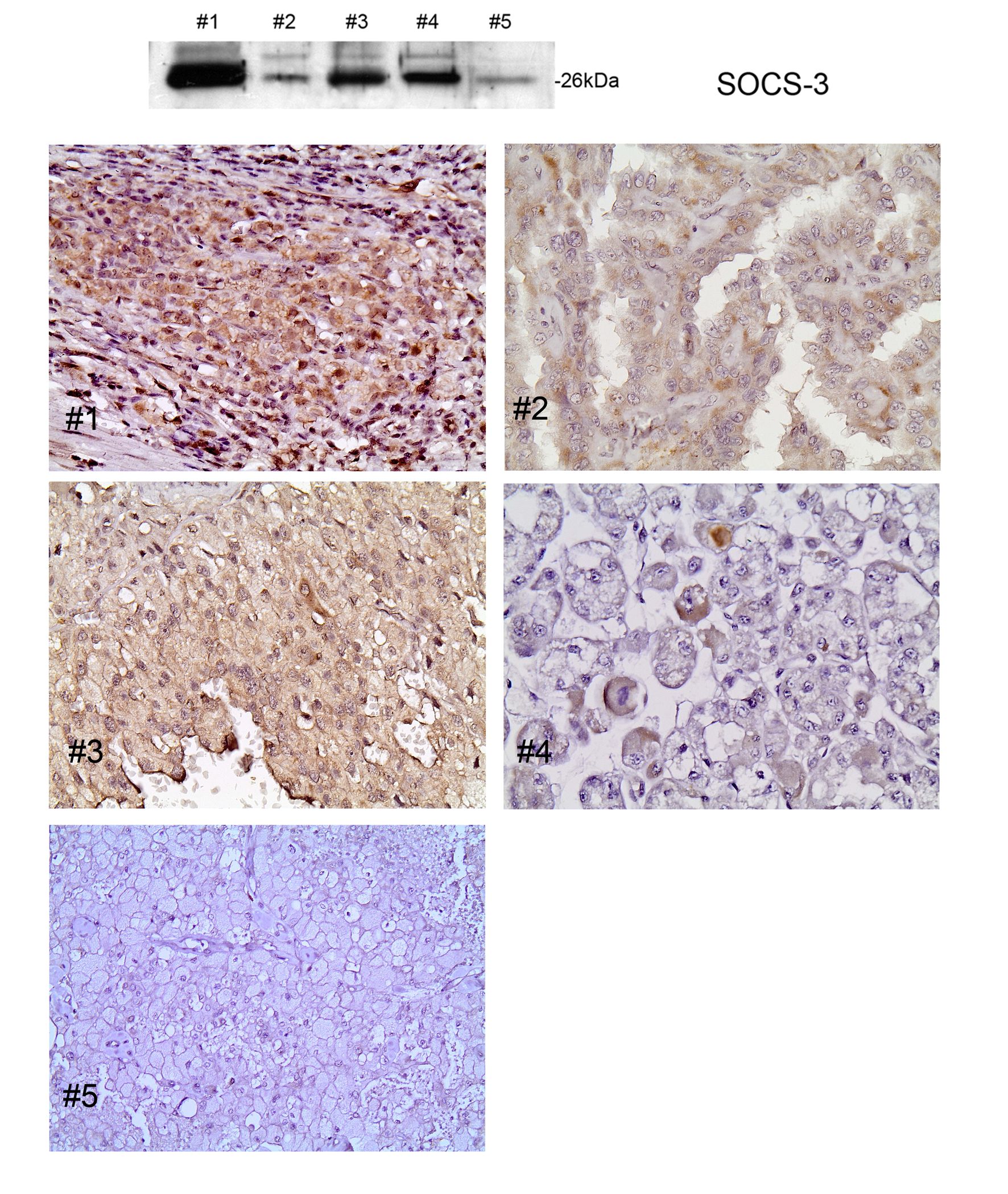

Supplement: Additional file 4 — SOCS-3 protein levels in fresh-frozen tissue specimens by Western blot and immunohistochemical expression of SOCS-3 in formalin-fixed tissue in the same five cases (#1, 4: Clear cell RCCs, #2: Papillary RCC, #3, 5: Chromophobe RCCs). Western immunoblotting validated the results of immunohistochemistry. [file 1471-2407-14-149-S4.tiff]

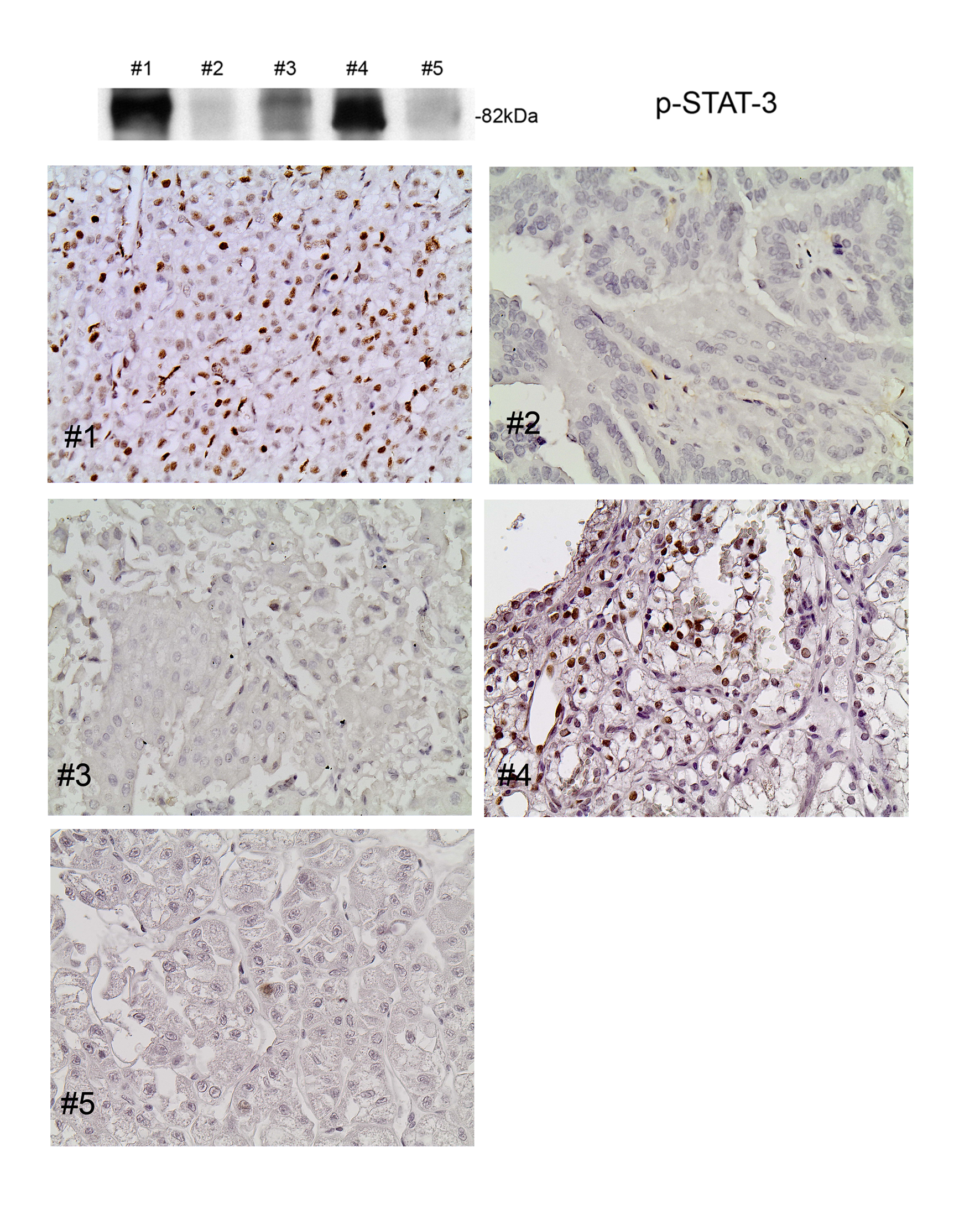

Supplement: Additional file 5 — p-STAT-3 protein levels in fresh-frozen tissue specimens by Western blot and immunohistochemical expression of p-STAT-3 in formalin-fixed tissue in the same five cases (#1, 4: Clear cell RCCs, #2: Papillary RCC, #3, 5: Chromophobe RCCs). Western immunoblotting validated the results of immunohistochemistry. [file 1471-2407-14-149-S5.tiff]
